# Clinical Application of Dynamic Contrast Enhanced Perfusion Imaging by Cardiovascular Magnetic Resonance

**DOI:** 10.3389/fcvm.2021.768563

**Published:** 2021-10-29

**Authors:** Russell Franks, Sven Plein, Amedeo Chiribiri

**Affiliations:** ^1^School of Biomedical Engineering and Imaging Sciences, King's College London, London, United Kingdom; ^2^Leeds Institute of Cardiovascular and Metabolic Medicine, University of Leeds, Leeds, United Kingdom

**Keywords:** myocardial perfusion imaging (MPI), cardiovascular magnetic resonance (CMR), quantitative perfusion, coronary artery disease, dynamic contrast enhance magnetic resonance, first-pass perfusion MRI

## Abstract

Functionally significant coronary artery disease impairs myocardial blood flow and can be detected non-invasively by myocardial perfusion imaging. While multiple myocardial perfusion imaging modalities exist, the high spatial and temporal resolution of cardiovascular magnetic resonance (CMR), combined with its freedom from ionising radiation make it an attractive option. Dynamic contrast enhanced CMR perfusion imaging has become a well-validated non-invasive tool for the assessment and risk stratification of patients with coronary artery disease and is recommended by international guidelines. This article presents an overview of CMR perfusion imaging and its clinical application, with a focus on chronic coronary syndromes, highlighting its strengths and challenges, and discusses recent advances, including the emerging role of quantitative perfusion analysis.

## Introduction

Myocardial perfusion imaging (MPI) plays a central role in the diagnosis, management, and risk stratification of patients with coronary artery disease (CAD) and is recommended by international guidelines (1). Unlike angiographic imaging, which provides anatomical data on the patency of major epicardial coronary arteries, MPI offers information on the downstream effects of epicardial coronary stenoses, as well as the function of the coronary microcirculation (2). Whilst there are a several non-invasive imaging modalities capable of MPI, cardiovascular magnetic resonance (CMR) is unique in its ability to provide high-resolution myocardial perfusion data alongside global and regional biventricular function, assessment of myocardial infarction, and without need for ionising radiation. This article presents an overview of the CMR method of dynamic contrast enhanced (DCE) perfusion imaging.

## Dynamic Contrast Enhanced Perfusion Imaging by CMR

**Figure 1:**
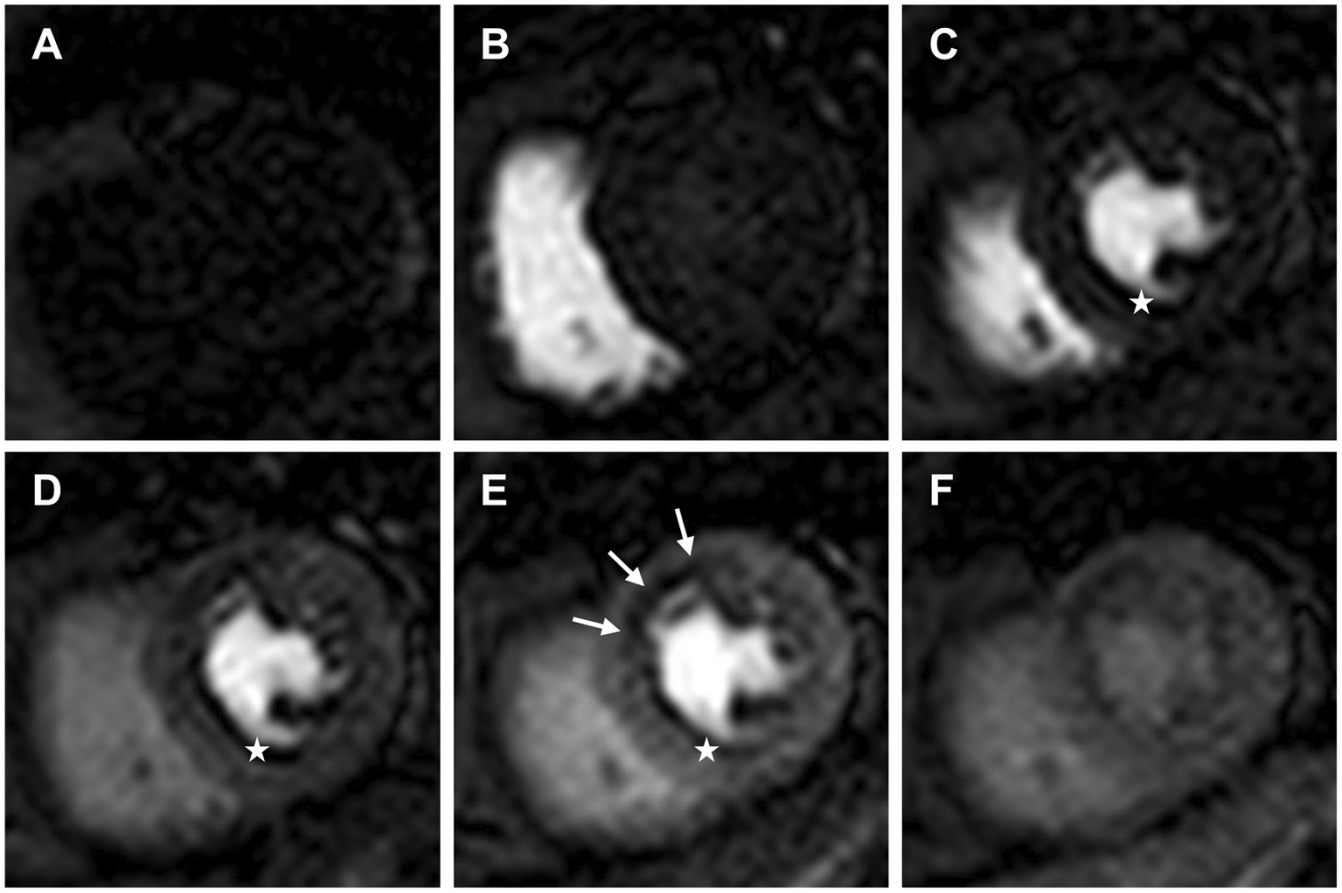
Dynamic contrast enhanced perfusion CMR tracks and displays the first passage of an injected contrast agent bolus through the heart. Mid ventricular slice **(A)** pre-contrast arrival; **(B)** contrast arrival in the right ventricle; **(C)** contrast arrival in the left ventricle (LV); **(D)** contrast arrival in the LV myocardium; **(E)** maximal myocardial contrast between remote myocardium and the subendocardial region of relative hypoperfusion (white arrows); **(F)** second pass and redistribution of the contrast agent. White stars identify a dark rim artefact, which can be seen on arrival of contrast in the LV prior to myocardial contrast enhancement.

DCE perfusion imaging is designed to track and display the first passage of a contrast agent (CA) bolus through the myocardium during maximal coronary vasodilation, and often during resting conditions (Figure 1) (3).

**Figure 2:**
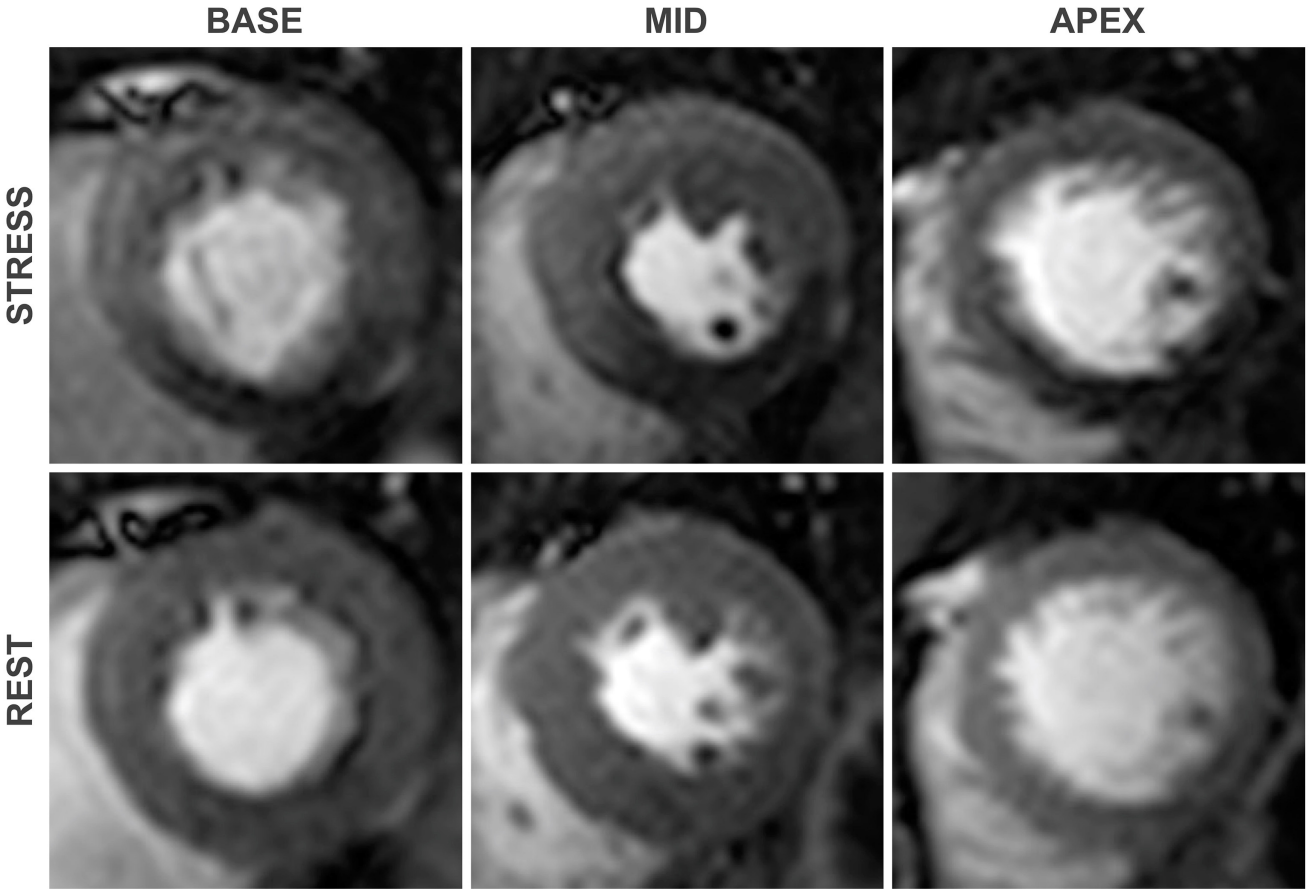
First-pass perfusion images at pharmacologically induced stress **(top)** and at rest **(bottom)**. An inducible perfusion defect is seen in the Inferior and inferoseptal segments when compared with the remote segments, consistent with flow limiting disease in the right coronary artery. As is typical of perfusion defects secondary to obstructive coronary stenoses, contrast hypoenhancement is most pronounced in the subendocardial region.

The technique relies upon the heterogeneity of contrast perfusion in myocardium supplied by obstructed vs. unobstructed coronary arteries. Flow limiting stenoses blunt the augmentation of myocardial perfusion during hyperaemia, manifesting as a relative perfusion defect at stress, which is not seen at rest, compared with myocardium subtended by unobstructed coronary arteries (Figure 2) (4). Maximal coronary vasodilation is typically achieved with an intravenous adenosine infusion or a bolus injection of the adenosine receptor agonist regadenoson. Both adenosine and regadenoson produce coronary vasodilatation by their agonistic action on the A2a receptors found in coronary smooth muscle and endothelial cells, inducing hyperpolerization of smooth muscle and release of nitric oxide (5). The phosphodiesterase enzyme inhibitor dipyridamole can also be used to induce coronary vasodilation. Dipyridamole inhibits cyclic adenosine monophosphate degradation and blocks cellular reuptake of adenosine, thereby increasing the circulating concentration of endogenous adenosine (6).

### Basic Principles

The clinical feasibility of tracking the first-pass of contrast through the myocardium with CMR was first demonstrated by Atkinson et al. in 1990 who used an inversion recovery gradient echo (GRE) sequence to track the first-pass of CA through rodent and human hearts (2, 7). Following an intravenous injection of CA, multiple (typically 3 short-axis) images of the heart are acquired, each with a different anatomical location and cardiac phase, which remain constant across sequential cardiac cycles (8). On arrival of the CA to the myocardium, the paramagnetic gadolinium chelate interacts with water molecules within the extracellular space, reducing T1 relaxation times and thus increasing signal intensity on a T1 weighted image. Areas of relative hypoperfusion therefore appear hypointense in comparison to well-perfused myocardium (4). In contemporary perfusion sequences, T1-weighting of images is typically achieved by use of a 90° saturation recovery (SR) radiofrequency pulse. A 180° inversion recovery pulse can generate greater contrast between normal and hypoperfused myocardium but is limited by longer imaging times and sensitivity to heart rate variations and miss-triggers, which can result in incomplete magnetisation recovery and signal intensity variation. Thus, saturation-prepared sequences are the current standard (2, 4). Preparation pulses are typically non-selective in order to reduce myocardial sensitivity to through-plane motion as well as achieve uniform contrast enhancement in the left ventricular (LV) blood pool. Whilst use of a single shared SR preparation offers increased efficiency, typically, separate SR preparations are used for each imaging slice to ensure uniform image quality (4).

To ensure adequate coverage of the 16 standard AHA segments, guidelines recommend a minimum acquisition of 3 short-axis myocardial slices, in addition to a minimum spatial resolution of 3 × 3 mm (3, 9). In order to accurately display changes in signal intensity over time, imaging should ideally be acquired for consecutive R-R intervals during the first passage of the contrast bolus (3). During pharmacologically induced stress, the R-R interval can be short and thus rapid data acquisition is needed to meet these high spatial and temporal demands. A standard spatial resolution of 2–3 mm can be achieved with a fast read-out sequence, such as fast GRE, balanced steady state free procession (bSSFP) or hybrid echo planar imaging, combined with spatial under-sampling (2). Increasing data sampling speeds further can be used to achieve even higher in-plane spatial resolution (1–2 mm) and/or greater spatial coverage. Conventional spatial-under-sampling techniques, such as parallel imaging, are limited to ~2-fold acceleration owing to a significant signal to noise (SNR) penalty above this acceleration level. However, under-sampling in both the spatial and-temporal domain can significantly increase data acquisition speed without compromising on either SNR or temporal resolution (10). Higher in-plane spatial resolution (1–2 mm) can reduce endocardial dark-rim artefact and improve the image quality and diagnostic accuracy for the detection of CAD including in single-vessel and multivessel disease (10, 11). If the spatio-temporal acceleration is used to increase spatial coverage, additional short-axis myocardial slices or even 3-dimensional (3D) myocardial perfusion data for whole-heart coverage can be acquired. 3D perfusion CMR is highly accurate to detect CAD as defined by invasive coronary physiology, however, any clinical benefit over conventional 3 short-axis high-resolution myocardial slices remains unclear (12, 13). Use of multiband radiofrequency pulses for simultaneous multi-slice data acquisition has been proposed as a strategy to increase spatial coverage whilst maintaining in-plane spatial resolution, however, this method still awaits clinical validation in patients with CAD (14).

In the clinical setting, perfusion CMR is performed at either 1.5 Tesla (T) or 3T field strengths, with 1.5T being more widely available. A major advantage of perfusion CMR at 3T is the superior SNR that can be obtained. The higher field strength also improves contrast enhancement, and importantly, offers improved diagnostic accuracy for the detection of single vessel and multivessel CAD (15, 16). Despite the overall image quality being superior at 3T (10), the associated increased field inhomogeneities heighten the sensitivity to susceptibility artefacts (17). Use of a GRE readout as opposed to a bSSFP readout is therefore preferred at 3T to minimise this undesirable consequence (18).

### Artefacts

An in-depth review of imaging artefacts is beyond the scope of this review, however, two types of artefact are of particular importance in DCE perfusion CMR imaging and warrant brief discussion. Despite high data acquisition speeds, DCE perfusion imaging is susceptible to both in-plane and through-plane motion, which can be cardiac or respiratory related, and results in image artefacts (4, 19). This can be exacerbated by a high respiratory rate during pharmacologically induced stress. In-plane motion can be reliably corrected in-line after data acquisition (20), however, through-plane motion remains problematic and highlights the importance of patient education and focus on gentle controlled breathing during the acquisition of stress images. Another important artefact in DCE perfusion CMR imaging is the subendocardial dark-rim artefact. There are several causes of dark-rim artefacts, the most common being Gibbs ringing, cardiac motion and magnetic field inhomogeneities resulting from the strong paramagnetic properties of a gadolinium based CA arriving in the heart (19). The dark-rim artefact is of particular importance as it can mimic inducible perfusion defects and reduce diagnostic accuracy. However, dark-rim artefacts do have features which enable an experienced reader to differentiate them from true perfusion defects. Typically, unlike true inducible perfusion defects, dark-rim artefacts appear on arrival of contrast in the LV blood pool, lead to a signal reduction compared with baseline (pre-contrast), are usually only one pixel wide, and most frequently appear in the phase encoding direction (21). An example of a dark-rim artefact is shown in Figure 1.

## Qualitative Stress Perfusion CMR

Qualitative stress perfusion CMR is one of the most robust non-invasive methods for the detection of CAD (22). The visual assessment of myocardial contrast enhancement during the first-passage of CA enables detection of regions of relative hypoperfusion. Comparison of myocardial perfusion is made between endocardial and epicardial regions as well as between myocardial segments. Significant inducible perfusion defects are more severe at the subendocardium, appear on the arrival of CA to the myocardium, are more than 2 pixels wide, and must persist beyond the peak myocardial enhancement. Furthermore, for a perfusion defect to be significant for ischemia, it should be present during stress but not at rest (if available) and, in the context of CAD, have a distribution consistent with one or more coronary territories (21). A transmural gradient of perfusion, with more severe hypoperfusion in the subendocardial layers, is usually observed in the involved segments (21, 23). Perfusion imaging is read alongside the corresponding late gadolinium enhancement (LGE) imaging, with matching LGE-perfusion defects being considered negative for inducible ischemia (21, 24).

There are numerous single centre and multi-centre studies demonstrating the accuracy of qualitative stress perfusion CMR. In a large meta-analysis by Jaarsma et al. data was pooled from 22 studies that evaluated qualitative stress perfusion CMR against anatomical luminal stenosis on invasive coronary angiography (ICA) and found a patient level sensitivity and specificity of 90 and 74%, respectively (25). Using invasive fraction flow reserve (FFR) as the reference standard, a more recent meta-analysis by Kiaos et al. pooled 6 studies and found sensitivity and specificity of 90 and 85%, respectively (26). More important for guiding revascularisation decision making is the ability of stress perfusion CMR to accurately detect ischemia at the level of the perfusion territory. In 2013 Ebersberger et al. evaluated 116 patients with suspected or known CAD with qualitative stress perfusion CMR at 3 Tesla and found a high diagnostic accuracy for detecting diseased vessels (defined by invasive coronary angiography with FFR) with an area under the receiver operator characteristic curve (AUC) of 0.93, sensitivity of 89% and specificity of 95% (27). Similar high diagnostic accuracies have also been reported by others (23, 28, 29).

The accuracy of qualitative stress perfusion CMR has been extensively compared with other non-invasive MPI modalities. In 2008, the MR-IMPACT study was the first multicentre multivendor study to demonstrate non-inferiority of stress perfusion CMR to the then clinical standard single-photon emission computerised tomography (SPECT) for the detection of CAD in 42 patients against coronary angiography (lumen stenosis > 50%) with AUCs of 0.86 and 0.75 for CMR and SPECT, respectively (30). The subsequent larger MR-IMPACT II study with 515 patients across 33 centres found superior sensitivity of perfusion CMR over SPECT (0.67 vs. 0.59) but inferior specificity (0.61 vs. 0.72) (31). In 2011 the single centre CE-MARC trial was the first large scale prospective comparison of CMR vs. SPECT for the detection of CAD on ICA (>70% stenosis) and found superior sensitivities (87 vs. 67%) and similar specificities (83 vs. 83%) for CMR vs. SPECT (32, 33). In 2015, a meta-analysis by Takx et al. compared the diagnostic accuracy of perfusion CMR with other non-invasive MPI techniques and found perfusion CMR had a similarly high diagnostic performance to positron emission tomography (PET) and computerised tomography (CT) perfusion and superior performance to SPECT and myocardial contrast echocardiography. This analysis, which included 15 perfusion CMR studies with 798 patients, found a pooled sensitivity and specificity for perfusion CMR of 0.89 and 0.87, respectively, at the patient level and 0.87 and 0.91 at the vessel level (22). It is noteworthy that this meta-analysis included CMR studies analysed qualitatively as well as quantitatively.

Multiple retrospective and prospective trials have demonstrated the prognostic value of qualitative stress perfusion CMR for risk stratification of patients with known or suspected CAD (34, 35). A large meta-analysis of 15 pooled studies evaluating 7,606 patients with known or suspected CAD undergoing stress perfusion CMR found a positive stress perfusion CMR was associated with an annualised event rate of 4.9% compared with only 0.9% in those with a negative study (35). Consistent with this, and its high diagnostic performance, stress perfusion CMR is an effective gatekeeper to invasive evaluation and management of patients with angina. In 2016, the CE-MARC II trial in patients with suspected angina found initial investigation by CMR resulted in a lower probability of unnecessary invasive coronary angiography than the since updated UK National Institute for Health and Care Excellence (NICE) guidelines–directed care, with no increase in adverse events (36). More recently, the multi-centre MR-INFORM trial demonstrated stress perfusion CMR can be used with the same efficacy and safety as invasive FFR in the initial management of patients with stable angina and risk factors for CAD (37). The study randomly assigned 918 patients to either a perfusion CMR scan or invasive coronary angiography with FFR, and found non-inferiority of the CMR strategy for the composite primary outcome of death, non-fatal myocardial infarction, or target-vessel revascularization within 1 year. In addition, the CMR based strategy was associated with a lower incidence of coronary revascularisation. Using stress perfusion CMR as a gate-keeper prior to ICA is also a cost effective approach in patients at intermediate risk of obstructive CAD (38).

### Challenges of Qualitative Stress Perfusion CMR

In addition to its strengths, qualitative stress perfusion CMR also has its challenges:

(1) Operator training - Perfusion data interpretation is subjective and as such is dependent on operator training and experience. The high diagnostic accuracies reported in the literature mostly come from experienced academic CMR centres with expert readers. A study from Villa et al. demonstrated that the level of reader training is the main determinant of diagnostic accuracy in the identification of CAD. They found Level 3 readers to have an 83.6% diagnostic accuracy compared with 65.7 and 55.7% for level 2 and level 1 readers, respectively (39).
(2) Balanced ischemia, multi-vessel disease and ischemic burden - Evidence of inducible myocardial ischemia is associated with adverse prognosis (35). Furthermore, prognosis worsens as the ischemic burden increases, and only revascularisation of flow-limiting coronary stenoses is associated with improved outcomes (40, 41). Therefore, accurate detection and quantification of the myocardial ischemic burden is of paramount importance when stratifying patient risk and considering coronary revascularisation. As aforementioned, qualitative stress perfusion CMR relies upon the heterogeneity of myocardial contrast perfusion associated with the presence or absence of flow-limiting coronary stenoses. In situations of global ischemia, such as three vessel disease (3VD) or microvascular dysfunction (MVD), there is often an absence of normally perfused reference myocardium and hence qualitative assessment can be challenging. The detrimental impact of “balanced ischemia” on diagnostic accuracy is well-documented in SPECT with up to 20% of patients with 3VD being incorrectly reported as normal (42), and as few as 29% of patients having perfusion defects reported in all coronary territories despite angiographically proven 3VD (43). Perfusion CMR has superior spatial resolution to SPECT (typically 1.5–3 vs. 12–15 mm) and is able to overcome this limitation to some extent, however, the diagnostic accuracy of qualitative assessment remains lower in patients with multivessel CAD and MVD (44–47). Kotecha et al. reported a diagnostic accuracy for qualitative stress perfusion CMR at 1.5 Tesla of 40 and 48% for correct classification of 3-vessel and 2-vessel CAD, respectively, as proven with invasive coronary angiography and FFR (48). Using a high-resolution perfusion CMR sequence, Motwani et al. demonstrated a diagnostic accuracy of 57% for detecting perfusion defects in all coronary territories in patients with angiographically proven 3VD (49). Rahman et al. recently reported a qualitative stress perfusion CMR sensitivity of 41% and specificity of 83% with an AUC of 0.60 to detect MVD defined by invasive physiology (44).
(3) Prior coronary artery bypass grafting (CABG) - Evaluation of myocardial perfusion in patients with prior CABG is challenging. These patients frequently have complex multivessel disease, established myocardial infarction, and extensive collateralisation (50, 51). Increased contrast dispersion, delayed contrast arrival at the myocardium (52), and the variability of flow dynamics associated with bypass grafts (53) add to the complexity and likely contribute to the reduced diagnostic accuracy of qualitative perfusion CMR compared with patients without prior CABG (24, 50). In the largest study to date of 110 patients with prior CABG (and 236 with previous percutaneous coronary intervention (PCI)), Bernhardt et al. reported a sensitivity and specificity of 73 and 77%, respectively, for detecting obstructive angiographic disease in patients with prior CABG, while in patients with previous PCI, a sensitivity and specificity of 88 and 90% were reported (24).

## Quantitative Perfusion CMR

Analysis of myocardial and LV signal intensity (SI)-time curves from DCE perfusion CMR enables quantitative and semi-quantitative analysis of myocardial perfusion, which has been proposed to offer a solution to some of the challenges of qualitative assessment (54).

### Semi-Quantitative Myocardial Perfusion CMR

**Figure 3:**
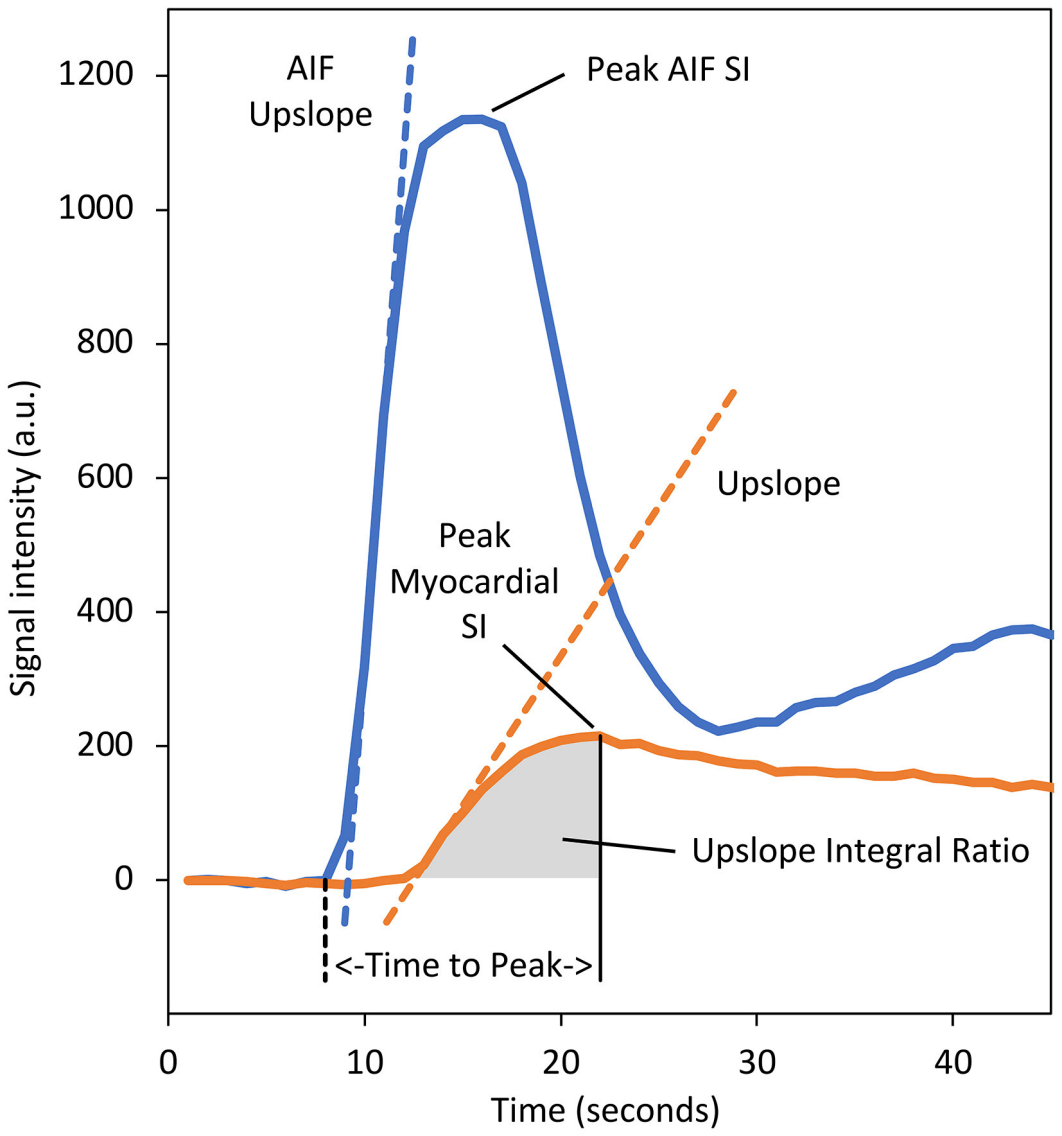
Baseline corrected signal intensity (SI)–time curves for myocardial tissue (orange) and the arterial input function (AIF) (blue) sampled from the left ventricular (LV) blood-pool. Various semi-quantitative perfusion parameters are demonstrated. The dashed orange line represents the “upslope” parameter and denotes the maximal rate of myocardial contrast enhancement. Division of the myocardial upslope by the equivalent AIF upslope (dashed blue line) defines the “upslope index,” which normalises for the haemodynamic conditions and enables comparison between stress and rest, and calculation of a myocardial perfusion reserve index. The area under the myocardial tissue curve from the arrival of contrast at the myocardium to the time of peak enhancement defines the “upslope integral ratio.” The area under the myocardial curve from contrast arrival to the time of peak AIF enhancement has also been used to define this parameter. The “time to peak” myocardial enhancement is measured from the arrival of contrast in the LV blood pool (dashed black line) to the time of peak myocardial enhancement. The “contrast enhancement ratio” parameter is not displayed as this requires an uncalibrated baseline (defined as SI_peak_−SI_baseline_)/SI_baseline_ (55). a.u., arbitrary units.

Whilst the field is moving toward absolute perfusion quantification, prior to the recent technical developments that made full quantification of myocardial perfusion possible, various semi-quantitative measures of myocardial perfusion were proposed. These methods describe the characteristics of the myocardial SI-time curves without attempting to estimate absolute myocardial blood flow (MBF). The most commonly used of these is the maximal myocardial “upslope” parameter, but others including “upslope integral ratio,” “contrast enhancement ratio (CER),” and the “time to peak” have also been evaluated (Figure 3) (21, 54, 55). Since myocardial perfusion is driven by systemic arterial perfusion, semi-quantitative parameters are dependent upon the underlying haemodynamic conditions and can be normalised to enable comparison of rest and stress values, as well as a comparison between individuals (54).

Underlying haemodynamic conditions are partially reflected by the shape of the arterial input, which can be measured from the LV blood pool. Division of the myocardial parameter by the equivalent arterial input function (AIF) parameter serves to normalise perfusion parameters and defines a perfusion index (PI). The ratio of the normalised stress and rest perfusion indices defines a myocardial perfusion reserve index (MPRI), a non-invasive surrogate for coronary flow reserve (CFR) and an index of the functional severity of a coronary lesion (54, 56, 57). It is noteworthy that this method of PI normalisation represents a heuristic approach, and one which has been shown to under-estimate perfusion reserve when compared against microspheres. A more accurate MPRI requires the PI to be normalised by the analogous AIF parameter as well as the time delay between the foot of the tissue curve and peak tissue enhancement (58).

Perfusion dyssynchrony has previously been proposed for the identification of hemodynamically significant CAD, as defined by FFR, and is based on the analysis of the variance of the time to peak across the LV myocardium (59).

Semi-quantitative parameters have been validated against microspheres and coronary angiography for detection of CAD with a high diagnostic accuracy (60–62). A recent meta-analysis of 6 studies using semi-quantitative analyses of myocardial perfusion at the territory level found pooled sensitivity of 77% and specificity of 84% (63). However, there are a number of drawbacks to semi-quantitative perfusion reserve indices:

(1) The only modest gains over qualitative assessment come at the expense of a significant time penalty necessary for processing the perfusion data (64).
(2) When validated against microspheres, semi-quantitative parameters underestimate MBF at flow rates above 1.5 ml/g/min. This can cause underestimation of PIs and MPRI in healthy myocardium where typical stress MBF rates exceed 2 ml/g/min (55).
(3) MPRI from different semi-quantitative perfusion parameters tend to have different thresholds to identify myocardial ischemia and their magnitude cannot be directly compared to that of an invasively measured coronary flow reserve (54).
(4) MPRI requires the acquisition of DCE perfusion imaging during stress and rest, partially conflicting with current international imaging guidelines, which are moving away from the routine acquisition of rest imaging in a quest to reduce CMR scan duration (3).
(5) MPRI is unable to distinguish between a state of globally reduced stress MBF with normal resting MBF (for example multivessel epicardial coronary disease), and a state of globally preserved stress MBF but increased resting MBF (for example hypertension or aortic stenosis). Both clinical scenarios result in a diminished global MPRI. Only by quantifying absolute MBF in millilitres per gramme of myocardium per minute (ml/g/min) during rest and maximal hyperaemia can we differentiate these two very different clinical scenarios (54).

### Quantitative Myocardial Perfusion CMR

Measurement of absolute MBF in ml/g/min is possible through the application of tracer-kinetic models to the perfusion data. Myocardial perfusion reserve (MPR), a useful indicator of the significance of coronary artery stenoses, is defined as the ratio of MBF at stress and rest (57).

Several different methodologies for perfusion quantification have been developed. The technical aspects of the various approaches are beyond the scope of our review, however, in brief the methods can be broadly divided into two distinct groups; “tracer-kinetic model dependent” and “tracer-kinetic model independent” (54). Model-dependent methods, of which there are numerous, make the assumption that the tissue structures can be divided into distinct compartments, typically an intravascular and interstitial compartment, and use complex mathematical equations to describe the contrast exchange occurring between these compartments. Tracer-kinetic models are applied to perfusion data beyond the first pass of contrast and can infer knowledge on the permeability surface area product and intravascular volumes in addition to MBF (54). The accuracy of the measurements is dependent on assumptions made with respect to parameters such as signal saturation, relaxivity of contrast agent, baseline T1 and T2 values in the myocardium and blood, homogeneity of the magnetic and RF excitation fields, blood heamatocrit and others (65).

Tracer-kinetic model-independent approaches are centred around the central volume principle, which dictates that MBF can be measured from knowledge of the contrast transit times through the vascular system (66). This value can be estimated from the transfer function, obtained by normalising the myocardial SI curves by the AIF curve using a deconvolution operation. The initial amplitude of the transfer function is proportional to MBF (67). The transfer function is, in practise, obtained using a forward modelling approach that involves the use of different mathematical models and data fitting procedures (67). One of the most widely used and validated deconvolution technique employs the use of a Fermi function to constrain the transfer function to fit the likely behaviour of an intravascular tracer (54, 67).

Quantitative perfusion (QP) measurements require the existence of a linear relationship between CA concentration and measured SI. However, the relationship between these parameters becomes non-linear at higher contrast concentrations due to saturation of the T1-weighted contrast enhancement and T2^*^ effects (68, 69). This phenomenon is more frequently observed in the AIF where CA concentration is highest. Any underestimation of the AIF will result in an overestimation of MBF with a magnitude relative to the magnitude of the saturation effect (54). Signal saturation must therefore be avoided or corrected for accurate MBF measurements. Use of a lower CA dose can avoid signal saturation in the blood pool but would provide inadequate contrast-to-noise for myocardial assessment. Proposed solutions include; (1) a dual-bolus acquisition; (2) a dual-sequence acquisition; and (3) retrospective correction using calibration curves (54). Whilst calibration curves can be generated from knowledge of the sequence parameters and the pre-contrast T1 measurements, this is a somewhat cumbersome approach to correct blood-pool signal saturation, and minor changes in the perfusion sequence parameters can necessitate the need for re-calibration (70). The dual bolus approach measures the AIF from a dilute CA pre-bolus, which maintains the linearity of SI to CA concentration in the blood pool. The pre-bolus is followed by a neat CA bolus for myocardial assessment (71, 72). As the dilution ratio is known, the AIF SI-time curve can be rescaled (and time shifted) prior to perfusion quantification (54). This technique has been validated against microspheres for measurement of MBF (55, 73) and has clinical validation against PET and invasive FFR for the detection of flow limiting CAD (23, 74, 75). However, clinical implementation of the dual bolus technique is onerous owing to the need for multiple injections and longer sequence acquisition times (72).

A dual sequence acquisition uses a single bolus of neat contrast but acquires an addition imaging slice with a short saturation delay, low resolution and reduced T1-weighting (69). This low resolution slice enables measurement of the AIF without blood pool signal saturation. Higher resolution perfusion slices are acquired following the low-resolution image and during the same R-R interval, permitting myocardial and AIF assessment within the same cardiac cycle (69, 76, 77). When a dual sequence approach is employed, and prior to quantification of MBF, SI-time curves must be converted to correspond to CA concentrations (69). A comparison of MBF estimates in pigs found a good correlation between dual-bolus and dual-sequence methods (77). The dual sequence method has been validated against invasive coronary physiology and PET, and is becoming the method of choice for quantitative CMR perfusion owing to its easier integration within the clinical workflow (78, 79).

Another important consideration prior to perfusion quantification is the intrinsic spatial variations in receive-coil sensitivity, which can produce SI variation across the myocardium and lead to inaccuracies when quantifying MBF. This can be corrected for by acquisition of proton-density weighted maps. Alternatively, coil sensitivity can be estimated from pre-contrast images and corrected for by dividing the myocardial signal by its pre-contrast value (54).

### Automation of Perfusion Quantification by CMR

**Figure 4:**
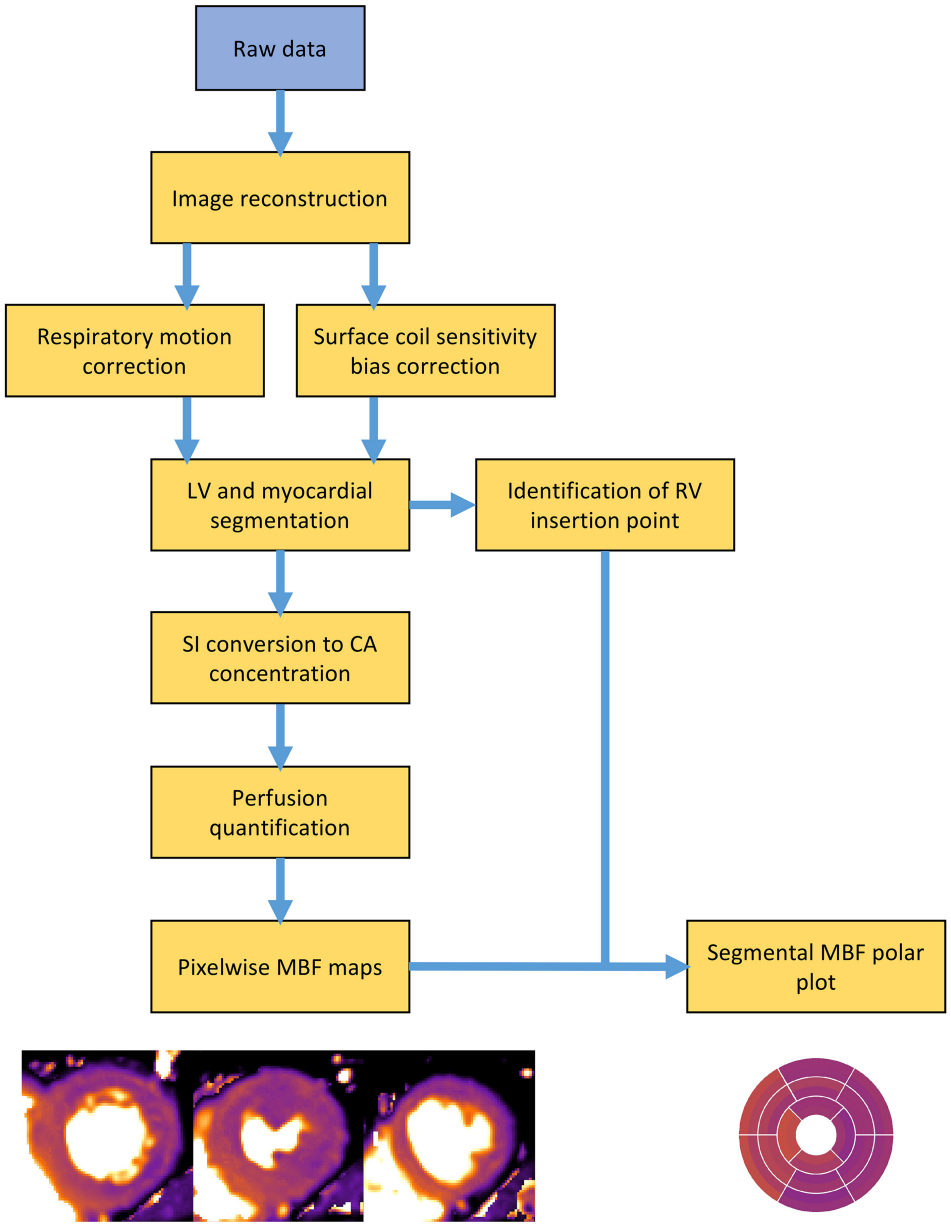
Overview schematic outlining the processing steps involved in a typical perfusion quantification pipeline. LV, left ventricle; RV, right ventricle; SI, signal intensity; CA, contrast agent; MBF, myocardial blood flow.

The quantification process is complex and requires multiple data processing steps (Figure 4). Perfusion images must first be reconstructed from the raw data. The dynamic image series require correction for respiratory motion in addition to correction for coil sensitivity bias (80, 81). Segmentation of the left ventricle and myocardium is required to enable extraction of the AIF and myocardial tissue curves. A point of reference, typically the superior RV insertion point, must be identified to enable standardised AHA cardiac segmentation (9). If a dual sequence approach has been employed, SI data requires conversion to CA concentration. Only at this stage are quantification models applied to the perfusion data to calculate MBF (81). Until recently, these multiple processing steps required time-consuming and laborious manual input, which restricted the application of quantitative perfusion CMR from mainstream clinical practise. However, recent developments in quantification pipelines now enables full automation of the quantification process and generation of pixel-wise perfusion maps either offline, or in-line and within minutes of data acquisition (79).

In 2017, Kellman et al. presented a fully automated quantification pipeline, utilising a dual-sequence approach for derivation of the AIF and a blood tissue exchange model for quantification of MBF. All reconstruction and processing steps were implemented in-line within the opensource Gadgetron software framework and pixel-wise perfusion maps were output within minutes of data acquisition (69, 82). In the same year this approach was validated against PET with good agreement between perfusion values (78), and later demonstrated good repeatability in a study of healthy volunteers, with within subject coefficient of variations between 8 and 12% for both rest and stress MBF measurements (20). In 2019, Kotecha et al. validated the same automated perfusion quantification pipeline against invasive coronary physiology and found high diagnostic accuracy with an AUC of 0.90 for detecting of functionally significant epicardial coronary disease (79). Knott et al. recently demonstrated a strong prognostic value of the same automated pipeline (83). Other fully automated perfusion quantification pipelines have since been developed. Using a two-compartment exchange model for perfusion quantification, Scannell et al. demonstrated highly accurate MBF values from an automated deep-learning based pre-processing pipeline when compared with manual pre-processing (84). Using model-constrained deconvolution, Hsu et al. demonstrated excellent correlation between perfusion values from fully automated and manual processing pipelines, as well as high diagnostic accuracy for the detection of CAD with AUCs between 0.86 and 0.93 for automated quantitative perfusion metrics (85).

### Clinical Value of Quantitative Perfusion CMR

**Figure 5:**
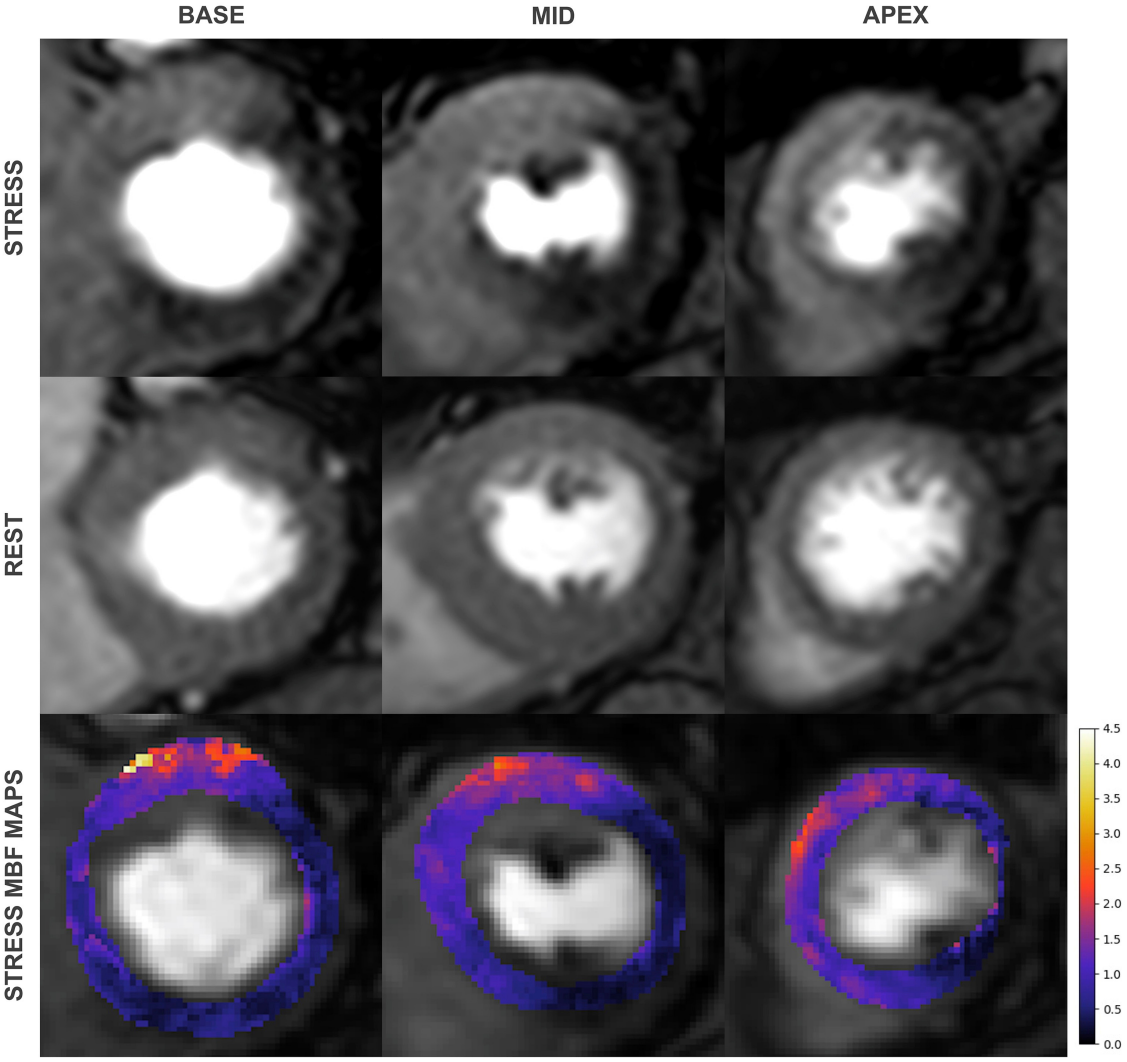
First-pass perfusion images during adenosine induced stress **(top)** and at rest **(middle)** with corresponding pixel-wise myocardial blood flow (MBF) maps **(bottom)** from a 79-year old man with significant 3 vessel disease on invasive coronary angiography. Qualitative assessment identifies inducible perfusion defects in the left circumflex and right coronary artery territories but only perfusion mapping identifies 3 vessel disease.

Several clinical advantages of QP CMR have been demonstrated over qualitative assessment:

(1) The diagnostic accuracy of QP CMR for the detection of CAD is at least equivalent to a level 3 experienced reader, thus offering an observer independent solution to smaller centres that may lack the experience and volume of the larger academic CMR laboratories. A study from Villa et al. found the diagnostic accuracy of QP CMR at the patient level was similar to the qualitative report of a level 3 trained reader and superior to a level 2 trained reader (QP: 86.3%, qualitative level 3: 83.6%, qualitative level 2: 65.7%) (39). This is consistent with a sub-study of the CE-MARC trial that compared stress MBF, MPR, and qualitative assessment (by expert readers in academic centres) and found no difference in diagnostic accuracies (86).
(2) In multivessel coronary disease QP CMR has superior diagnostic accuracy for the detection of CAD at the vessel level (Figure 5). A study by Kotecha et al. with a cohort of 151 patients (95 patients with multivessel disease defined by invasive angiography with FFR), found stress MBF to have superior diagnostic accuracy than qualitative perfusion CMR to identify 3-vessel (87 vs. 40%) and 2-vessel disease (71 vs. 48%) but similar accuracy for the detection of single vessel disease (71 vs. 71%) (48). In line with these findings, QP CMR also enables a more accurate estimation of the myocardial ischemic burden in cases of multivessel disease. In a study of 41 patients, Patel et al. found qualitative assessment was unable to differentiate the ischemic burdens of single-vessel and three-vessel CAD (21 vs. 31%, *p* = 0.26) unlike myocardial perfusion reserve (MPR), which found a significant difference (25 vs. 60%, *p* = 0.02) (45). A similar advantage of QP CMR is also seen in patients with MVD, in whom global ischemia is often present without a region of reference myocardium. A recent study by Rahman et al. of 75 patients with non-obstructed coronary disease, found MPR had a diagnostic accuracy of 79% for the detection of MVD (defined by invasive physiology), significantly superior to qualitative assessment, which had a diagnostic accuracy of 58% (44). Similar findings were reported by Kotecha et al. who found stress MBF had 71% sensitivity, 70% specificity and an AUC of 0.73 for detecting MVD. This study went on to demonstrate that global stress MBF thresholds could be used to non-invasively differentiate 3-vessel coronary disease form MVD (79).
(3) Another potential advantage of QP CMR is its ability to correct for scarred myocardium. The pixel-wise nature of perfusion quantification enables pixel-wise exclusion of LGE from the analysis. This was demonstrated in a study by Villa et al. who fused myocardial perfusion reserve maps with LGE maps to quantify microvascular ischemia in patients with hypertrophic cardiomyopathy. They demonstrated that not accounting for LGE leads to a significant overestimation of the ischemic burden (87). This combined QP and LGE analysis has also been demonstrated feasible in patients with ischemic cardiomyopathy in whom high scar burdens are often present and revascularisation decision making is complex (88). The prognostic value of such an approach to predict myocardial recovery post revascularisation remains unclear.
(4) There is emerging data that QP CMR enables improved patient risk stratification. In 395 patients with suspected CAD and a median follow up of 460 days, Sammut et al. found that the ischemic burden as measured by MPR provided incremental prognostic value to qualitative assessment (89). In another study of 1,049 patients with suspected or known CAD and a median follow up of 605 days, Knott et al. found stress MBF and MPR were both independently associated with death and major adverse cardiovascular events (83).
(5) Unlike semi-quantitative analysis, fully quantitative analysis has the potential to improve clinical workflow by automated post processing, and potential to reduce scan time by acquiring only stress data. There is some evidence that the accuracy of stress MBF is not enhanced by measurements of MBF at rest (79, 90), however, this remains a contentious issue as evidence to the contrary also exists, particularly in patients with MVD (44, 74, 91).

### Quantitative Perfusion CMR and PET

Cardiac PET enables highly accurate measurements of myocardial perfusion, particularly when tracers with linear or near linear extraction are used, and is currently the clinical standard for the non-invasive quantification of myocardial perfusion (78, 92). Recently, perfusion quantification by CMR has been validated against PET with good agreement between perfusion measures (78). As compared to cardiac PET, CMR offers the advantage of higher spatial resolution, wider availability, lower costs, and freedom from ionising radiation. However, where-as the volumetric acquisition of cardiac PET allows for full heart coverage, 3D whole-heart perfusion imaging by CMR remains confined to a research tool and clinical CMR perfusion imaging is typically planned to sample the 16 standard AHA myocardial segments using 3 short-axis slices (3, 13).

## Future Directions

DCE stress perfusion CMR has made significant strides over the past 2 decades to become an accurate, well-validated, and safe non-invasive method for assessing the functional significance of CAD (26). Advances in data acquisition methods appear promising and high-resolution full LV coverage is likely to be achieved in the coming years (14). The development and validation of QP CMR offers incremental diagnostic and prognostic value, particularly in patients with advanced CAD (48, 89). Advances in artificial intelligence technology are likely to play an increasingly important role in the clinical interpretation of perfusion maps (83). However, for the widespread use of QP CMR outside of the academic institutions, cross vendor standardisation and regulatory approval for the use of in-line myocardial perfusion quantification is required.

## Conclusion

Stress perfusion CMR is a well-validated and guideline-backed non-invasive tool for the assessment and risk stratification of patients with CAD. Qualitative analysis has high diagnostic accuracy and prognostic value when performed by experienced readers. Contemporary, fully automated perfusion quantification pipelines can provide an accessible, reliable, observer independent analysis with superior diagnostic and prognostic performance, particularly in patients with complex multivessel CAD and MVD.

## Author Contributions

RF: writing—original draught, review and editing. SP and AC: writing—review and editing. All authors contributed to the article and approved the submitted version.

## Funding

The authors acknowledge financial support from: The British Heart Foundation [PG/18/71/34009 and TG/18/2/33768]; The Department of Health via the National Institute for Health Research (NIHR) comprehensive Biomedical Research Centre award to Guy's & St Thomas' NHS Foundation Trust in partnership with King's College London and King's College Hospital NHS Foundation Trust; The NIHR Cardiovascular MedTech Co-operative; Wellcome/EPSRC Centre for Medical Engineering [WT 203148/Z/16/Z].

## Author Disclaimer

The views expressed are those of the authors and not necessarily those of the NHS or funding bodies. The funding bodies did not have a role in the writing of this manuscript.

## Conflict of Interest

The authors declare that the research was conducted in the absence of any commercial or financial relationships that could be construed as a potential conflict of interest. The handling editor declared a shared research group with one of the author AC at time of review.

## Publisher's Note

All claims expressed in this article are solely those of the authors and do not necessarily represent those of their affiliated organizations, or those of the publisher, the editors and the reviewers. Any product that may be evaluated in this article, or claim that may be made by its manufacturer, is not guaranteed or endorsed by the publisher.
